# Penetrating Gun-shot Wound of the Eye

**Published:** 1872-08

**Authors:** J. W. Southworth

**Affiliations:** Toledo, O.


ART. III.—*Penetrating gunshot-wound of the Eye, lodgment of a small bird shot in the sclerotica opposite the point of entrance.* Reported by J. W. SOUTHWORTH, M. D., Toledo, O.

J. C. aged 17 while out fowling July 4th, 1868 was accidentally shot by a comrade, several shots struck on his cheek and back, some lodging just beneath, others in, the skin. One missle striking the outer aspect of the globe just above its horizontal meridian, about a line from the margin of the cornea; penetrating the sclerotic coat, at least, as evidenced by the presence in the orifice of the dark pigmented tissues underlying.

The first physician that examined it was of the opinion that the shot had not penetrated the organ, but had fallen out of the wound after partially burying itself in the sclerotica and prescribed accordingly.

On the 9th of July the patient consulted Dr. N. H. Kimball of Adrian, Mich. The eye was considerally inflamed, not very painful nor had he experienced much pain. Vision was obscure and intense photophobia was complained of. The organ was extremely sensitive to the touch and there was opacity of the crystaline lens which was greatest at its upper half. The wound appeared as before stated. Perfect rest, and exclusion from light and an active antiphlogistic treatment was adopted for the next few days. On the 13th the inflammation had subsided sufficiently to admit of an examination with a probe, so as to determine positively the penetration of the missle into the interior of the eye. Patient was anesthetized with chloroform, but the strabismus incident to it, so interfered with the examination that he was allowed to regain consciousness. After which the patient held the eye still by voluntary effort and the probe passed in by its own weight, the point of it being visible through the pupilary aparture. It was gently raised so as to cause the end to impinge against the floor of the posterior chamber and drawn out, without producing any flinching of the patient, nor giving any evidence to the hand of the surgeon of contact with the shot which was unquestionally lodged in the organ.

The patient and father were apprised of the possible occurrence of sympathetic opthalmitis, and the necessity of prompt operative procedures, in case it should begin to develop.

July 16th, eye much improved, very little redness of conjunctiva, can see his hand held up to the light, but cannot count his fingers. No pain and but slight photophobia treatment, small dose of calomel, opii and tart. antimon. at bed time. Iod. potass grs. vii in solution ter die, with a mild astringent and sedative collyria. Aug. 1st, eye apparently well, with opacity of lens sufficient to prevent recognition of countenances at three feet. Discharged patient with instructions to report immediately on the occurrence of trouble in either eye.

Jan. 10th, 1869. Patient presents himself with a small rounded elevation of sclerotic coat at its inner aspect just opposite the cicatrix of former wound. It has a dark bluish color and it unquestionably caused by the shot burrowing or ulcerating its way to the surface. Patient could not see any better. The missle was left to work out its own salvation.

